# Recent Advances in Nose-to-Brain Gene Delivery for Central Nervous System Disorders

**DOI:** 10.3390/pharmaceutics17091177

**Published:** 2025-09-10

**Authors:** Flávia Nathiely Silveira Fachel, Angélica Salatino-Oliveira, Willian da Silva Carniel, Rafaela Zimmermann, Ursula Matte, Helder Ferreira Teixeira, Guilherme Baldo, Roselena Silvestri Schuh

**Affiliations:** 1 Programa de Pós-Graduação em Ciências Farmacêuticas, Faculdade de Farmácia, Universidade Federal do Rio Grande do Sul, Porto Alegre 90610-000, RS, Brazil; willian.carniel@ufrgs.br (W.d.S.C.); rafaela.zimmermann@ufrgs.br (R.Z.); helder.teixeira@ufrgs.br (H.F.T.); roselena.schuh@ufrgs.br (R.S.S.); 2 Programa de Pós-Graduação em Genética e Biologia Molecular, Universidade Federal do Rio Grande do Sul, Porto Alegre 91501-970, RS, Brazil; asdeoliveira@hcpa.edu.br (A.S.-O.); umatte@hcpa.edu.br (U.M.); gbaldo@hcpa.edu.br (G.B.); 3 Laboratório Células, Tecidos e Genes, Serviço de Pesquisa Experimental, Hospital de Clínicas de Porto Alegre, Porto Alegre 90410-000, RS, Brazil

**Keywords:** brain delivery, CNS delivery, nasal administration, nanotechnology, nucleic acids

## Abstract

The nasal route represents a promising non-invasive technique for the direct delivery of nucleic acids to the central nervous system (CNS) disorders, effectively bypassing the blood–brain barrier. This route offers several advantages, including ease of administration, enhanced patient compliance, rapid therapeutic onset, and increased availability. Nonetheless, challenges such as mucociliary clearance, enzymatic degradation, and the low permeability of cell membranes to large molecules remain obstacles to the effectiveness of this approach. To address these limitations and achieve targeted nose-to-brain delivery with optimized therapeutic outcomes, various technological solutions have been explored, such as nanotechnology-based delivery systems and mucoadhesive formulations. These innovations aim to enhance the permeability of the nasal mucosa, extend the residence time of therapeutic agents in the nasal cavity, and improve overall treatment effectiveness. While the nasal gene delivery to the brain is still relatively new, it holds considerable potential for expanding treatment options for a range of CNS disorders. In this context, this review examines the anatomy and physiology of the nasal route, the mechanisms of biomolecule transport from nose to brain, the potential of gene delivery vectors, key preclinical advancements, and clinical perspectives for the nasal delivery of nucleic acids in CNS disorders.

## 1. Introduction

Gene therapy has emerged as a promising treatment for various central nervous system (CNS) disorders, such as neurodegenerative diseases, brain tumors, and genetic neurological conditions [1,2]. However, delivering biomolecules to the brain remains a significant challenge due to the restrictive nature of the blood–brain barrier (BBB), which prevents the crossing of many therapeutic agents, including nucleic acids [3]. Traditional methods, such as intracerebral injections or systemic administration, are often invasive and inefficient, highlighting the need for alternative, non-invasive drug delivery strategies [4].

In recent years, the nasal route has become a promising alternative for delivering genes directly to the CNS, offering a non-invasive way to bypass the BBB through nose-to-brain pathways [5]. This route offers several benefits, including ease of self-administration, good tolerability, and minimal discomfort, making it particularly suitable for repeated use in long-term treatment regimens [6]. Furthermore, nasal delivery enables the rapid onset of action due to the highly vascularized nature of the nasal mucosa, which allows quick absorption and transport of drugs to the brain. It can also avoid first-pass metabolism, improving bioavailability, reducing required doses, and minimizing potential side effects [7,8].

The nasal mucosa, as a highly perfused tissue, supports the absorption of both small and large molecules, including nucleic acids (e.g., plasmid DNA, mRNA, siRNA). Despite these advantages, the effectiveness of nose-to-brain delivery is limited by challenges such as low permeability, mucociliary clearance, and enzymatic degradation in the nasal cavity, which can reduce nucleic acid retention and availability [2]. To overcome these limitations and improve the efficiency of nose-to-brain gene delivery, various technological approaches for the nasal route have been explored, such as nanotechnology-based delivery systems, mucoadhesive strategies (such as cationic polymers and lipids), in situ gels, and penetration enhancers [2,3,6,9].

In this context, this review examined the physiology of the nasal route, including the transport mechanisms of biomolecules. It highlighted its potential as a promising strategy for delivering nucleic acids from the nose to the brain. It also emphasized the role of gene delivery vectors in ensuring the effective delivery of nucleic acid-based therapies. Additionally, this study focused on both preclinical advancements and clinical perspectives in gene therapy for nose-to-brain delivery, aiming to treat CNS disorders.

## 2. Nasal Route

The nasal route is widely used to achieve local effects in the treatment of tissue inflammation, allergic rhinitis, and nasal congestion, utilizing typical drugs such as antihistamines, glucocorticoids, or decongestants, which are available in various formulations, including nasal sprays, solutions, drops, gels, and other topical preparations. A significant advantage is that the drug is administered locally, resulting in a rapid improvement of symptoms while reducing the dose administered. This targeted delivery minimizes potential side effects and enhances therapeutic outcomes [10,11].

In addition to local applications, the nasal route has gained increasing importance in the systemic delivery of drugs, particularly for biologic agents such as peptides and nucleic acids, which are unstable in the gastrointestinal tract and prone to high first-pass metabolism when taken orally. However, nasal administration enables these agents to bypass the gastrointestinal system, resulting in higher bioavailability and a faster onset of action. Compared to oral administration, nasal delivery offers the advantages of reduced first-pass metabolism, increased absorption through the nasal epithelium, and longer-lasting therapeutic effects [10,11].

A particularly intriguing aspect of nasal drug delivery is its potential for direct access to the CNS. The anatomy of the nasal cavity provides a unique pathway for drugs to reach the brain by bypassing the BBB via the nose-to-brain pathway. This is achieved through the olfactory and trigeminal nerve pathways, which connect the nasal cavity directly to the brain. The BBB, which usually protects the CNS from harmful substances, also poses a significant challenge for delivering therapeutic agents to treat neurological disorders. By leveraging the nasal route, drugs and biomolecules can circumvent this barrier, offering a promising strategy for the treatment of CNS conditions [1,3,6,12].

Despite these advantages, the efficacy of gene delivery via the nasal route can be influenced by several factors related to nasal physiology and anatomy. Pathological and physiological conditions affecting the nasal mucosa, such as inflammation or congestion, can reduce the absorption and effectiveness of gene delivery. Additionally, adverse effects associated with nasal gene delivery may include irritation of the nasal mucosa, local inflammation, and immune responses triggered by genes or vectors. In some cases, repeated or high doses may lead to mucosal damage or chronic inflammation. These side effects can compromise the safety and effectiveness of the gene delivery system. Understanding these potential risks, alongside their benefits, is crucial for optimizing nasal gene delivery systems and ensuring their success in local, systemic, and nose-to-brain applications [2,4,5,12,13].

The nasal cavity has a volume of 25 cm^3^ and measures 12–14 cm from the nostrils to the nasopharynx. It plays a key role in humidifying the air, regulating temperature, and removing harmful microorganisms. Located between the roof of the mouth and the skull base, it is supported by the ethmoid bones above and by the ethmoid, maxillary, and inferior conchae bones on the sides. The cavity is divided into two halves by the nasal septum, with each half referred to as a nasal cavity. These are further segmented into three parts: the nasal vestibule, atrium, and turbinates (which include inferior, middle, and superior) [3,4,6,9,14].

The respiratory region, which includes the inferior turbinate, is the primary site for the entry of drugs and biomolecules into the systemic circulation due to its large absorption surface area (~160 cm^2^), presence of microvilli, extensive vascularization, and high permeability. In contrast, the olfactory region is a small area made up of stratified hair cells, located in the middle and upper parts. Although its surface area is limited (12.5 cm^2^), the olfactory epithelium provides a pathway for drug delivery directly to the brain, bypassing the BBB, through the olfactory and trigeminal pathways. Notably, the olfactory neuroepithelium is distinguished by its unique regenerative capacity and ability to communicate with the external environment [3,4,6,9,14].

Each nasal cavity is lined by an epithelium mainly consisting of pseudostratified columnar epithelial cells, goblet cells, basal cells, and mucous and serous gland cells, all of which are connected by intercellular junctions. On top of this epithelium lies a layer of nasal mucus, serving as a physical barrier that protects cells and influences nasal drug absorption. Secretory glands and goblet cells produce mucus through granules filled with mucin, a glycoprotein that determines its viscosity. This mucus forms a thin, viscous layer (approximately 5 µm) on the epithelium, composed of water and other substances, including glycoproteins, proteins, electrolytes, salts, lipids, and additional components. The high viscosity of mucus is mainly due to mucin. It produces about 1.5 to 2 L daily, primarily serving as a protective layer against pathogenic and allergenic microorganisms [3,4,6,9].

In this sense, key obstacles to nucleic acids delivery via the nasal route include poor absorption of these large polar biomacromolecules due to low nasal membrane permeability, as well as the limited dose per nostril that can be administered, due to the small volumetric capacity of the nasal cavity (a maximum dose of 0.4 mL for humans) [15].

Moreover, many epithelial cells are lined with microvilli and cilia that help transport, remove, and replace mucus, aiding its movement toward the nasopharynx. A significant limiting factor is mucociliary clearance, which reduces absorption through the nasal mucosa by regularly removing and renewing the mucus layer at a rate of approximately 5–6 mm/min. Additionally, the presence of mucus can hinder drug absorption by binding the drug or biomolecule to mucin, the main negatively charged protein in mucus [3,6,7,9,15].

Various pathological and physiological conditions affecting the nasal mucosa can directly affect drug absorption and efficacy. For instance, physiological changes caused by allergy, irritation, and inflammation, often worsened by itching and sneezing, may influence how drugs are absorbed. The nasal mucosa itself contains a variety of enzymes, such as reductases, monooxygenases, transferases, and proteolytic enzymes, which can break down drugs and thus reduce their absorption [4,7,15,16].

## 3. Nose-to-Brain Nucleic Acids Delivery

The primary challenge in delivering nucleic acids to the brain for the treatment of CNS disorders is the BBB. The BBB is a thin network of blood vessels with tightly joined endothelial cells, which isolates the brain from the circulatory system and shields it from harmful substances such as toxins and bacteria. Nose-to-brain drug delivery is currently one of the methods that provide direct access to the brain, bypassing the BBB [2,5,15].

**Figure 1:**
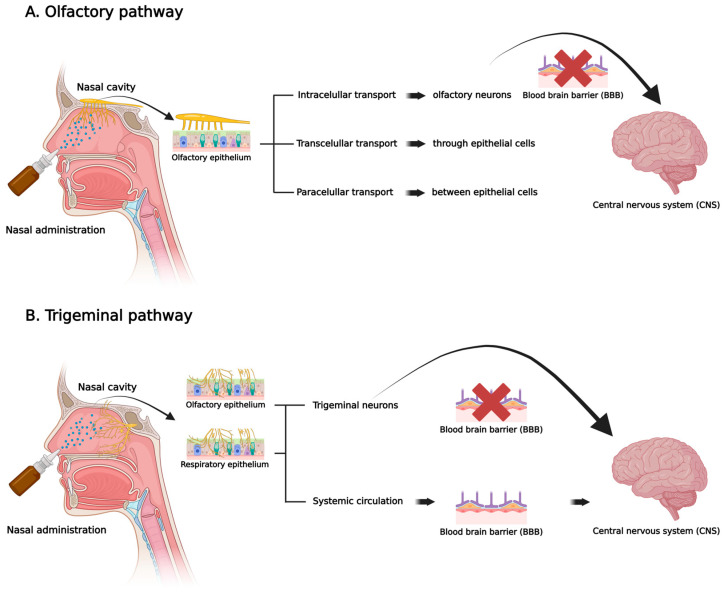
Scheme of nose-to-brain drug molecules and biomolecules delivery via olfactory (**A**) and trigeminal (**B**) pathways, illustrating possible mechanisms of nucleic acids-based therapeutics absorption through the nasal cavity to reach the central nervous system (CNS) (Created in BioRender. Carniel, W. (2025) https://BioRender.com/jdjm3bu, accessed on 30 July 2025).

Nevertheless, the precise mechanism by which nucleic acids are transported to the brain via the nasal route remains unclear, with ongoing debate among researchers. Nonetheless, it is generally thought that the main pathways, the olfactory and trigeminal nerves, primarily utilize passive diffusion as the primary means of absorption from the nose to the brain [2,10,17,18]. Consequently, when the nucleic acid-based therapy is administered nasally, it can be transported to the CNS via the olfactory or trigeminal pathways, through the respiratory or olfactory epitheliums, following mainly transcellular, intracellular, and paracellular absorption mechanisms as depicted in Figure 1.

In the nasal epithelium, cells are linked through various junctions, including tight junctions. These junctions are impermeable to biomacromolecules, such as nucleic acids; however, due to the ongoing turnover of neuronal and basal cells, they can become permeable to these molecules. When these junctions open, they can facilitate paracellular transport. Conversely, transcellular transport via receptor-mediated endocytosis is the primary pathway for biomolecules, involving endogenous receptors such as the transferrin receptor, insulin receptor, low-density lipoprotein receptor-related protein, nicotinic acetylcholine receptor, insulin-like growth factor receptor, diphtheria toxin receptor, scavenger receptor cell type B, leptin receptor, and the neonatal Fc receptor. In summary, the physicochemical properties of biomolecules—such as molecular size greater than 300 Da and high hydrophilicity—can influence and modify the mechanism by which drugs are transported through the nasal mucosa [1,10,19].

In the olfactory pathway, the olfactory epithelium comprises three cell types: neuronal cells, progenitor cells, and supporting cells, all of which are connected by tight junctions. Neuronal cells transmit information to the brain and extend from the nasal cavity’s olfactory epithelium to the olfactory bulb in the CNS. This connection enables drugs administered directly into the nose to reach the brain via the olfactory nerve in the olfactory bulb, which then projects to various brain regions, including the piriform cortex, amygdala, and hypothalamus. Although the olfactory epithelium accounts for only about 3% of the human nasal cavity, nasal delivery remains a practical route because there are no synapses between the afferent pathway and the receptive elements of olfactory neurons [1,10,19,20,21,22].

In the trigeminal pathway, the trigeminal nerve (the fifth cranial nerve) has three branches: the ophthalmic nerve, the maxillary nerve, and the mandibular nerve. This pathway is the most significant route currently studied for nose-to-brain delivery. The ophthalmic and maxillary nerves innervate the nasal mucosa and transmit essential information from the nasal cavity to the CNS. This information is projected to the brainstem via the pons. It enters the forebrain through the cribriform plate, facilitating the entry of drugs into both the caudal and rostral brain regions. The pathway involves axonal transport through the trigeminal nerves, which innervate both the respiratory and olfactory epithelia [1,10,19,20,21].

Compared to the olfactory pathway, which directs the drug to the rostral part of the brain, the trigeminal pathway reaches both the rostral and caudal regions. This makes it challenging to determine whether, during nasal administration, the drug is transported to the rostral area through the olfactory pathway or the trigeminal pathway [1,10,19,20,21].

In summary, the nasal route offers a non-invasive, easily self-administered, highly vascularized, and immunologically active environment that is advantageous for the nose-to-brain delivery of nucleic acids. However, the efficiency of this delivery remains limited, with low volumes per administration and vectors predominantly targeting entry points such as the olfactory and trigeminal pathways. Several barriers impede successful transfection and transduction via the nasal route, including mucociliary clearance, enzymatic degradation of nucleic acids, and the low permeability of cell membranes to large, polyanionic DNA or RNA molecules. Moreover, immune surveillance can trigger inflammatory responses. Additional challenges include anatomical variations in the nasal cavity (both anatomical and physiological differences), as well as the impact of delivery devices on drug delivery outcomes in preclinical and clinical studies [4,6,8,19].

## 4. Nose-to-Brain Gene Delivery Vectors

Nucleic acid-based therapeutics can modulate gene expression both at the transcriptional and post-transcriptional stages. They include oligonucleotides, DNA aptamers, DNA plasmids, nucleosides, antisense RNA, small interfering RNAs, microRNA, and other related molecules [23]. However, unmodified, naked nucleic acids are especially vulnerable to nuclease cleavage or can provoke an immune response [2]. To enhance their stability, bioavailability, and cellular uptake, various viral and non-viral vectors have been developed. These vectors protect nucleic acids from nucleases and facilitate their transport into target cells and the nucleus. The design of gene delivery systems must be tailored to specific therapeutic targets and delivery routes [24].

### 4.1. Viral Transduction and Non-Viral Transfection

Viral transduction and non-viral transfection are critical steps in gene delivery, referring to the process by which foreign nucleic acids are introduced into host cells to achieve gene expression, deletion, or silencing. Nasal delivery, although promising, is particularly challenging due to the unique anatomical and physiological properties of the nasal cavity. Successful transfection or transduction is essential for achieving effective therapeutic outcomes, as it involves not only the delivery of genetic material to the target site but also the efficient internalization and expression within cells of the nasal epithelium, olfactory region, or even in CNS-related pathways via the olfactory and trigeminal nerves. Unwanted transduction and transduction may occur along the nasal route, and pathological conditions in the nasal cavity can reduce the efficiency of administration, necessitating careful design of gene delivery vectors to circumvent these limitations [2,3,25].

### 4.2. Viral Vectors

Viral vectors, such as lentivirus, herpes simplex virus, adenovirus, and adeno-associated virus (AAV), exhibit high transduction efficiency, resulting in sustained gene expression; however, they also pose significant safety concerns. Both adenovirus and herpes simplex virus are pathogenic in humans and can infect cells, causing various symptoms and diseases. Lentivirus, an RNA retrovirus, inserts its genetic material into the host genome, which carries a risk of insertional mutagenesis. Among these, AAV has become the most successful gene delivery system due to its high efficiency and stability, along with low immunogenicity and toxicity [26], making it a promising tool for advancing gene therapies [25].

It is essential to recognize that the nose-to-brain delivery of AAV has primarily been investigated in preclinical research. Still, the exact mechanisms and distribution patterns of AAV in the brain following nose-to-brain administration need further investigation to confirm this promising delivery route. Additionally, olfactory sensory neurons and trigeminal nerves, which are situated along the nasal pathway, can also be transduced, potentially leading to side effects. This concern can be mitigated by designing the AAV with a cell-type-specific promoter to avoid transduction along the nasal route. Moreover, conditions affecting the nasal cavity, such as upper respiratory tract infections, can influence the efficiency of nose-to-brain delivery. These factors must be carefully considered when implementing this method in preclinical or clinical studies [27].

### 4.3. Non-Viral Vectors

Non-viral vectors are gene delivery systems that offer the benefit of reduced immunogenicity; however, their transfection efficiency is typically low, requiring repeated administrations [28]. Various non-viral vectors are employed to deliver gene-editing tools, with many drugs utilizing non-viral carriers that have been FDA-approved, most notably those based on lipid nanoparticles (LNPs). Additionally, new non-viral carriers are actively being developed and utilized in the creation and clinical testing of novel drugs [24]. Currently, non-viral vectors used for nucleic acid nose-to-brain delivery offer a certain degree of safety and carrying capacity compared to viral vectors.

#### Nanostructures and Biomaterials

Nanotechnology-based systems have garnered attention due to their ability to overcome the limitations of naked nucleic acid delivery from the nose to the brain. Targeted delivery, utilizing ligands that bind to specific receptors expressed on the nasal epithelium, further enhances cell-specific transfection, making these non-viral vectors a promising option for delivering nucleic acids to the brain [29].

Nevertheless, the success of such systems in promoting biological activity with low toxicity depends not only on their structure but also on the choice of biomaterials. Essential properties for nanocarriers and their materials include biodegradability, biocompatibility, and stability [30], as well as the capacity to protect nucleic acids, transfect target cells, and facilitate cost-effective production [28]. It is also crucial to consider the size, charge, and hydrophobicity of the nanostructure, which can be optimized to improve biopharmaceutical and pharmacokinetic properties and reduce adverse effects. Notably, nasal administration provides a very short residence time for molecules, with a half-life clearance of 15–30 min for non-adhesive liquid and powder formulations [31].

**Figure 2:**
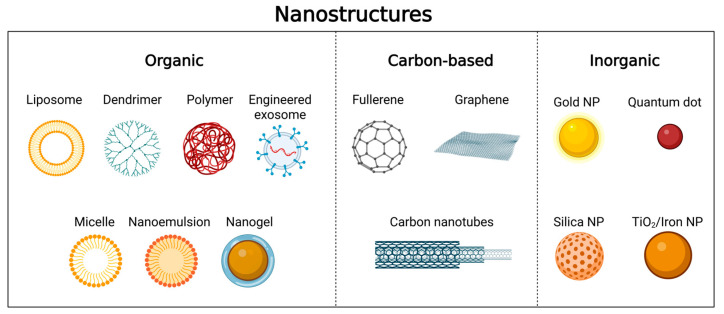
Compilation of the most common nanostructures used for delivering nucleic acids to the central nervous system (CNS) via nasal administration (Created in Biorender. Fachel, F. (2025) https://BioRender.com/4zs6d4h, accessed on 30 July 2025).

In this sense, smaller particles offer a larger surface area for interaction and absorption in the olfactory epithelium, thereby maximizing delivery to the target site [32]. Non-viral carriers, such as lipid nanoparticles, polymer-based carriers (polyplexes), dendrimers, and solid lipid nanoparticles, are widely used for the delivery of nucleic acids to the central nervous system (CNS) via nasal administration [17], as shown in Figure 2, which illustrates various nanostructures, including organic, carbon-based, and inorganic materials.

**Figure 3:**
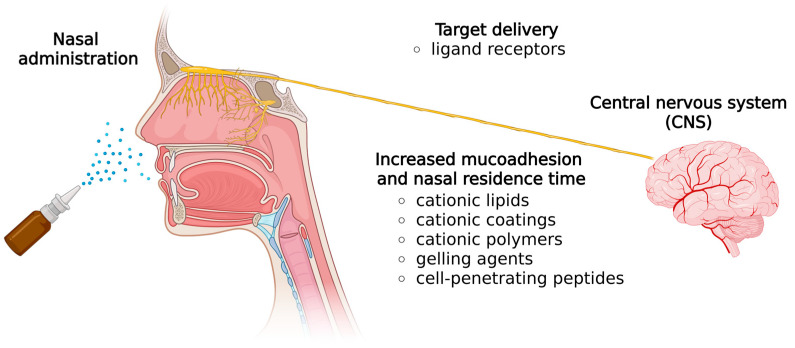
Common strategies to enhance nasal residence time and targeted delivery of nucleic acids to the central nervous system (CNS). (Created in BioRender. Fachel, F. (2025) https://BioRender.com/ueo1wx8, accessed on 30 July 2025).

Several technological strategies are employed to prolong the residence time of nucleic acids in the nasal cavity and enhance absorption, as shown in Figure 3. Non-viral mucoadhesive gene delivery strategies, such as cationic lipids and polymers, and in situ gelling agents, swell upon contact with the nasal mucosa, improving residence time and bioavailability [24]. Additionally, cell-penetrating peptides (CPPs) enhance the ability of these vectors to cross cell membranes [33].

Among lipid systems, liposomes are vesicles composed mainly of phospholipids, and genetic material can be adsorbed onto their surface or encapsulated in their cavity [34]. Liposomes enhance the stability of nucleic acids, reduce their hydrolysis rate, and extend their circulatory half-life [35]. A nanoemulsion is a liquid–liquid dispersion of two immiscible liquids stabilized by surfactants. Because of the high lipophilicity of the inner phase, nucleic acids typically adsorb onto the droplet surface. Similarly to liposomes, it usually requires the inclusion of positively charged molecules, such as cationic lipids or cationic polymer coatings, in the formulation [28].

Polymer nanoparticles typically consist of polymers, lipids, or a mix of both. Based on their components and preparation methods, nucleic acids can either be integrated into the structure for enhanced protection against degradation or attached to the surface [35]. When these systems include a polymer (typically water-insoluble), the term ‘nanoparticle’ can refer to both a completely polymeric core structure (nanosphere) and one with an oil core (nanocapsule) [36].

The key properties of nanostructures largely depend on the selected biomaterials. Typically, delivering nucleic acids requires a high association rate, effective transfection, the ability to functionalize (such as surface modifications), and an excellent safety profile. Additionally, for effective nasal delivery, mucoadhesive components help prolong residence time by reducing the clearance of nucleic acids from the nasal cavity. In parallel, nanotechnology-based systems—such as nanoemulsions—can also protect against enzymatic degradation in nasal secretions [37].

Regarding mucoadhesive formulations, it is well known that cationic lipids and polymers can interact with the negatively charged sialic acid groups of mucin [38]. Additionally, cationic materials are indispensable for associating with nucleic acids [39]. In this sense, DOTAP (*N*-[1-(2,3-dioleoyloxy)propyl]-N,N,*N*-trimethylammonium), DC-Chol (3-beta-[*N*-(dimethylaminoethane)-carbamoyl] cholesterol), DOTMA (*N*-[1-(2,3-dioleyloxy)propyl-N,N,*N*-trimethylammonium), and DDAB (didecyldimethylammonium bromide) are the most commonly used cationic lipids [28,30,40,41]. Similarly, PEI (polyethyleneimine) is a cationic polymer used for the same purpose. It can condense high-molecular-weight nucleic acids, improve stability, extend residence time, and prevent enzyme degradation [41,42]. However, their application in biological studies is often restricted because they interact with cell membranes, causing cytotoxicity. This toxicity can be mitigated through complexation with nucleic acids, as the negatively charged groups interact the positive charge of cationic lipids, resulting in lower interfacial charge values [43]. Moreover, the choice of mucoadhesive polymers and their ingredients is a key parameter that determines the rate and extent of nasal absorption.

Schuh and colleagues [44] administered nanoemulsions complexed with a plasmid encoding the IDUA (alpha-L-iduronidase) protein via nasal delivery, aiming to reach the brain for mucopolysaccharidosis type I (MPS I) gene therapy. These nanoemulsions, composed of DOPE (dioleoylphosphatidyl-ethanolamine), DOTAP, MCT (medium chain triglycerides), and DSPE-PEG (1,2-distearoyl-sn-glycero-3-phosphoethanolamine-polyethyleneglycol), were prepared using high-pressure homogenization. The resulting complexes effectively enhanced enzyme activity both in vitro and in vivo. In a second study, the same group developed cationic liposomal complexes carrying two plasmids—one with the CRISPR/Cas9 system and another with the *IDUA* gene—targeting the *ROSA26* locus for nose-to-brain delivery in MPS I mice [44]. This treatment modestly increased IDUA activity in the lungs, heart, and brain, indicating potential to prevent cognitive damage, supported by improvements seen in behavioral tests in the treated mice [45].

Azambuja et al. [46] administered a nasal delivery of a CD73siRNA-loaded cationic nanoemulsion containing DOTAP to a rat glioblastoma model. This nanoemulsion formed complexes with negatively charged siRNAs, facilitating their entry into cells and interaction with intracellular targets. As a result, CD73 was silent both in vitro and in vivo, leading to a 60% decrease in tumor size [46].

Dhaliwal et al. [47] used cationic liposomes containing GFP-mRNA to explore the nasal route for brain delivery. The liposomes, made of 1,2-dipalmitoyl-sn-glycero-3-phosphocholine (DPPC), DOTAP, and cholesterol, successfully transfected macrophages and stably expressed GFP protein. In vivo biodistribution revealed significantly higher GFP-mRNA expression—approximately 15%—in the brain compared to the naked mRNA group at 24 h after administration. The authors also noted that dose, frequency, and the timing of protein expression are key factors in determining CNS transfection efficiency. Therefore, delivering mRNA via cationic liposomes through the non-invasive nasal route can effectively target specific brain regions [47].

Cui and Mumper [48] developed cationic nanoparticles using a microemulsion precursor composed of the cationic surfactant cetyltrimethylammonium bromide (CTAB) and the nonionic surfactant Brij 78. They then adsorbed plasmid DNA (pDNA) onto the nanoparticle surfaces to explore their use as a delivery system for pDNA vaccines. When these pDNA nanoparticles were administered nasally to Balb/C mice, serum antigen-specific IgG levels increased by 18 to 28 times compared to naked pDNA. Additionally, a stronger proliferative response of splenocytes was observed after immunization with these nanoparticles, providing strong evidence for their potential in delivering pDNA via nasal vaccine [48].

In another study, cationic PEI (polyethyleneimine)-siBeclin1 nanoplexes were administered nasally to an HIV-infected mouse model by Rodrigues et al. [49]. Researchers detected siRNA in the glial cells of the prefrontal cortex and the cytoplasm of neurons at 4 and 24 h post-administration, with a notable 65% reduction in protein levels and no signs of toxicity. These findings suggest that nasal delivery can effectively target the central nervous system (CNS), offering a promising approach for gene silencing therapy [49].

Poly(acrylic acid)-based polymers, such as polyacrylate and polyacrylic acid (PAA), are utilized in nasal delivery systems due to their mucoadhesive properties. In addition to being anionic polymers, the thiolation of PAA enhances its mucoadhesion by creating disulfide bonds. Derivatives of PAA also include Carbopol and polycarbophil [50].

Vetter and colleagues [51,52] conducted two ex vivo studies using nasal mucoadhesive microparticles to deliver phosphorothioate antisense oligonucleotides (PTO-ODNs). These microparticles were coated with the mucoadhesive polymer polycarbophilic cysteine (PCP-Cys) or thiolated polycarbophil. The findings showed that these microparticles reduced clearance from the nasal cavity, increased contact time with the nasal mucosa, and offered high stability, improved ASO permeation, and controlled release. Notably, PTO-ODN incubated with thiolated polycarbophil/reduced GSH microparticles demonstrated a twofold increase in permeability. These results indicate that thiolated polycarbophil/reduced glutathione microparticles are a promising carrier for intranasal phosphorothioate ASO delivery. Both studies confirmed that this novel polymer protects against mucosal enzymes and enhances transport through the porcine nasal mucosa [51,52].

Naturally occurring polymers, particularly polysaccharides such as chitosan and alginate, have been extensively researched as components for nanocarriers [53,54,55,56]. Chitosan is a linear heteropolymer primarily obtained through the deacetylation of chitin in a basic environment, composed of repeating *N*-glucosamine and *N*-acetylglucosamine units. Its abundant free amino groups give it a positive charge, enabling it to interact with negatively charged nucleic acids [57]. Chitosan has been extensively utilized in pharmaceutical and medical applications due to its beneficial biological properties, including biodegradability and biocompatibility [53,58].

Perez et al. [59] used 1% *w*/*w* chitosan and poloxamer as mucoadhesive agents to develop thermosensitive mucoadhesive gels. They first formed 32P-labeled siRNA dendriplexes by electrostatically complexing siRNA with cationic dendrimers, then incorporated these into the gels. The dendriplexes showed decreased degradation by RNases and enhanced endocytic uptake. Intranasal delivery of these gels increased radioactivity in the brain. Mucoadhesive polymers extend the residence time of the loaded material, thus improving the nanoplatforms. To achieve higher brain radioactivity than intravenous dendriplexes or nasal naked siRNA, a combination of siRNA-dendrimer complexation, dendriplex incorporation into the gel, and two nasal doses was necessary [59].

In a study by Sava et al. [54], chitosan was used as a matrix-forming polymer for nanoparticles. Electrostatic interactions occurred between the positively charged amino groups of chitosan and the negatively charged phosphate groups of siRNAs. Additionally, Mangafodipir served as a cross-linking agent to stabilize the nanoparticle structure and protect siRNA from degradation. The divalent metal transporter may play a key role in the early endosomal processing of nanoparticles. Factors that enhance the production of effective nanocarriers for anti-HTT siRNA were identified and tested in a YAC128 mouse model of Huntington’s disease (HD). Four nanocarrier formulations were found to reduce HTT mRNA expression by at least 50%. Intranasal delivery of these siRNA-loaded nanoparticles offers a promising, safe, and effective method for lowering mutant HTT levels [54].

Poloxamers, also known as Pluronics, are nonionic triblock copolymers composed of polar polyethylene oxide blocks and non-polar polypropylene oxide blocks, which give the polymers amphiphilic and surface-active characteristics [60]. These hydrogels remain fluid at room temperature but solidify into a thicker gel at body temperature. This allows the in situ gelling system to stay at the topical site for an extended period, ensuring controlled and prolonged drug release [61].

Park and colleagues [62] created nasal delivery systems for plasmid DNA that utilize poloxamers (Pol) as in situ gelling agents, combined with polycarbophil (PC) or polyethylene oxide (PEO) as mucoadhesive agents. In vitro tests showed that Pol alone released plasmid DNA the fastest, whereas a formulation with 0.8% Pol/PEO released it the slowest. These formulations were administered nasally to mice, revealing that the AUC values for Pol/PC and Pol/PEO 0.4% were 10.9 and 9.6 times higher, respectively, than those of the saline control. This likely resulted from increased contact between the plasmid DNA and the nasal mucosa. The authors also proposed that poloxamer-based vehicles could form micelles that help attach plasmid DNA to the nasal surface, thereby enhancing its dispersibility and stability. Overall, the findings suggest that combining in situ gelation with mucoadhesive polymers can effectively and safely improve the nasal absorption and retention of nucleic acids [62].

Other methods involve using gelling polymers, such as hyaluronic acid, mPEG-PLA (a copolymer of poly(lactide) and poly(ethylene glycol)), and mPEG-PCL (a copolymer of poly(ε-caprolactone) and poly(ethylene glycol)). These can potentially increase the duration of nucleic acids in the nasal cavity.

Yang et al. [63] developed multifunctional core–shell nanomicelles coated with hyaluronic acid (HA) and encapsulating the cell-penetrating peptide DP7-C for siRNA delivery. These nanomicelles were administered nasally to rats and successfully transported siRNA to the CNS via the trigeminal nerve pathway within hours. Moreover, increased accumulation was observed at tumor sites, with no damage to the trigeminal nerves or nasal mucosa. Successfully delivering the formulation to GL261 tumor-bearing mice resulted in tumor growth inhibition, longer survival times, and smaller tumor volumes. The study suggests that these nanomicelles could be an effective intranasal delivery system for siRNAs in glioma treatment [63].

Kanazawa et al. [64] developed nanomicelles formulated using polyethylene glycol-polycaprolactone (PEG-PCL) bioadhesive copolymers conjugated with the cell-penetrating peptide Tat. Nasal administration of these nanomicelles to rats resulted in a ten-fold increase in siRNA levels in the brain. Two formulations were prepared: (i) siRNA targeting Raf-1 (siRaf-1) loaded into mPEG-PCL-Tat micelles [mPEG-PCL-Tat/siRaf-1 complexes], and (ii) siRNA with Raf-1 (siRaf-1) and camptothecin (CPT) co-loaded into mPEG-PCL-Tat micelles [CPT-loaded mPEG-PCL-Tat/siRNA complexes]. These were administered intranasally to assess their therapeutic effects in a rodent model of malignant glioma. Distribution studies revealed significantly higher siRNA concentrations in the brain compared to those achieved with intravenous injections. The average survival times were 16.6 days for untreated rats, 18.4 days for rats treated with naked siRaf-1, 20.4 days with mPEG-PCL-Tat/siRaf-1, 20.6 days with CPT-loaded mPEG-PCL-Tat/siControl, and 28.4 days with CPT-loaded mPEG-PCL-Tat/siRaf-1. The authors concluded that the cell-penetrating peptide-modified block copolymer enhances the delivery of siRNA and drugs to the brain via nasal administration, suggesting its potential use in clinical brain tumor and CNS therapy disorders [64].

There are several nanoparticulate delivery systems, such as lipid NPs, polymeric micelles, exosomes, and polymeric NPs, for nucleic acids delivery to the brain using functionalization strategies in Alzheimer’s disease (AD) research [65]. In this sense, Su et al. [66] developed mPEG-PLA nanoparticles loaded with miR-132, aiming to treat AD and cerebral ischemia. Since naked miRNA is easily degraded or expelled via mucous membranes after administration, a carrier is needed that guarantees stability, safety, and targeted delivery. Both mouse and rodent models of AD with ischemic brain injury received intranasal nanoparticle treatments. The distribution of WGA-NPs-miR132 in the APP/PS1 mouse model and MCAO rats was notably improved in the brain, demonstrating therapeutic effects, reduced nasal ciliary clearance, and better targeting to neurons. The authors concluded that nasal delivery of miRNA nanoparticles holds significant promise for treating neurodegenerative disease conditions [66].

Solid lipid nanoparticles (SLNs) are colloidal particles made of lipids that remain solid at room and body temperatures. The use of solid lipids instead of liquids is believed to enable controlled drug release, likely due to the smaller size of the solid lipid core compared to lipid systems containing liquid oils, such as emulsions. Similarly to other lipid vectors discussed earlier, cationic lipids help generate a positive charge at the particle surface [67,68], while small oligonucleotides are internalized into the solid matrix [69].

Rassu et al. [69] created SLNs for nose-to-brain delivery of BACE1 siRNA to treat Alzheimer’s disease, incorporating RVG-9R, a peptide that facilitates cell penetration and protects the oligonucleotide. These SLNs were produced via an emulsification-solvent evaporation process using a double emulsion (*w*/*o*/*w*) technique. After production, their surfaces were coated with chitosan to improve their mucoadhesive properties. In vitro tests using the Caco-2 cell line demonstrated that formulations containing nanoencapsulated oligonucleotides exhibited increased permeability. In contrast, those coated with chitosan showed decreased passage through the cell layer, accompanied by increased intensity [69].

Despite the recent rise in synthetic nanoparticles in nanotechnology, biological nanoparticles have been present in the environment for thousands of years. These naturally occurring particles are produced within biological systems and vary widely in size. The term “biological nanoparticles” covers a broad range of structures, including vesicles and macromolecules secreted by cells, such as exosomes, as well as intracellular components like magnetosomes. Exosomes are bilayer membrane structures composed of lipids and proteins, measuring between 30 and 150 nanometers in diameter. They are known to carry numerous proteins and nucleic acids, with an estimated content of about 100 proteins and 10,000 nucleotides [70,71].

Li et al. [72] developed exosomes derived from mesenchymal stem cells (MSCs) that were functionalized with the viral peptide RVG29 for siRNA delivery in the treatment of AD. They loaded the exosomes with siRNAs targeting BACE1 and Caspase-3, combined with the cationic polymer BAP (poly-[(2-methacryloyl)ethyl(p-boronic acid benzyl) dimethylammonium bromide]), creating a polyplex called REXO/BAP@siRNAs for siRNA complexation. Administered intranasally in 3xTg-AD mouse models, the exosomes significantly increased brain accumulation of Cy5-labeled siRNA, as shown by fluorescence. The treated mice outperformed saline controls in spatial learning and memory tests, demonstrating improved escape behavior. These outcomes closely resembled those of wild-type animals [72].

In summary, the main studies using nanocarriers and biomaterials as nose-to-brain gene delivery systems are summarized in Table 1. It is essential to highlight that choosing a nanocarrier for nucleic acid-based therapies requires a comprehensive evaluation of all formulation precursors and components, as well as assessing its tolerability. Additionally, crucial factors such as physicochemical properties, stability, cell and tissue uptake, biodistribution, and pharmacokinetic parameters must be carefully considered [73]. An ideal nanotechnology-based system for nucleic acid delivery should be able to both protect the genetic material and deliver it to the target, ensuring patient safety. Recent advances in biomaterials and nanotechnology will enable the development of new systems with enhanced capabilities for delivering nucleic acids via the nasal route.

## 5. Preclinical Advancements in Gene Delivery for CNS Disorders

Gene therapy research for various CNS disorders has explored nasal delivery as a strategy to overcome limitations of systemic administration [2,5,74]. This route has been investigated in preclinical studies as a promising method for delivering various nucleic acids to different brain regions involved in diverse CNS disorders.

Nose-to-brain delivery has been explored for many years, yet its underlying mechanisms remain not fully understood. Consequently, research efforts have focused on determining how transport occurs, as well as the processes involved in transport and distribution via this pathway. To our knowledge, the earliest report of brain transgene expression following intranasal administration of a non-viral DNA nanoparticle vector was published in 2014. That study demonstrated that single molecules of plasmid DNA encoding enhanced GFP (eGFP), compacted with 10 kDa polyethylene glycol (PEG)-substituted lysine 30-mers (CK30PEG10k), administered intranasally, resulted in widespread transfection and eGFP expression throughout the rat brain. The eGFP protein was detected two days after delivery, mainly near capillary endothelial cells along the rostral-caudal axis [75]. Recently, the same research group utilized a novel reporter plasmid, pUGG, which encodes an eGFP-GDNF fusion protein, to investigate the regional distribution, cell-type specificity, and temporal dynamics of transgene expression in rat brains following intranasal delivery. Both eGFP ELISA and counting eGFP-positive cells demonstrated that intranasal administration of these nanoparticles resulted in successful transfection and sustained transgene expression throughout the brain. Most transfected cells were found along capillary walls and are likely pericytes. The intranasal approach yielded significant, long-lasting transgene expression, peaking at one week and persisting for up to six months post-treatment [76].

Various nanocarriers have been explored to enhance intranasal delivery of nucleic acids to the brain. Sanchez-Ramos et al. [77] showed that intranasal instillation of manganese-loaded chitosan-matrix nanoparticles (mNPs) containing anti-GFP siRNA reduced GFP expression by at least 50% in four brain regions: the olfactory bulb, hippocampus, cerebral cortex, and corpus striatum in transgenic green mice. Moreover, mNPs carrying dsDNA for red fluorescent protein were successfully expressed in the corpus striatum and other areas after intranasal administration [77].

Petkova et al. [78] aimed to target the cerebral cortex and examined the effect of hyaluronidase-coated polyplexes (GCPH) to facilitate brain delivery via the nasal route. β-Gal DNA (GCP3)-loaded GCPH produced high β-Gal activity in the cortex when compared with both naked plasmid and lipofectamine lipoplexes, and was 10-fold less toxic than lipofectamine [78].

The transfection of the nerve growth factor-inducible (VGF) gene using modified chitosan nanomicelles was evaluated through both intranasal and intravenous delivery methods. VGF is essential for learning and memory, and it is involved in the pathophysiology of psychiatric disorders and neurodegenerative diseases [79]. Nasal administration of pVGF in polyplexes resulted in significantly higher brain protein expression compared to the intravenous delivery [80].

Various methods have been investigated to enhance brain-wide transfection and transgene expression via nose-to-brain delivery. Padmakumar et al. [81] investigated whether the minimally invasive nasal depot (MIND) technique could directly deliver an antagoNAT to the brain, which can derepress brain-derived neurotrophic factor (BDNF). They compared its effectiveness to that of direct invasive intracerebroventricular (ICV) injections. The MIND method reduces variability and addresses efficiency issues commonly encountered in standard nasal delivery [79]. BDNF plays a role in neuronal plasticity, dendritic growth, and neuronal survival, and has been suggested as a biomarker of neuroplasticity [82,83]. The authors found that depot implantation facilitated CNS distribution of ATs, resulting in significant and sustained BDNF upregulation, approaching 40% efficiency of ICV delivery [81].

### 5.1. Brain Injuries

Gene therapy shows promise for traumatic brain injury (TBI). Das et al. [84] studied chitosan and polyethyleneimine-coated magnetic micelles (CPMM) that delivered pCMV-tdTomato plasmids encoding the tRFP protein to rat brains after mild TBI. These CPMMs successfully crossed the BBB, and magnetofection enhanced the micelle concentration in the brain. tRFP expression was detected in the cortex, hippocampus, lung, and liver within 48 h of injury. The CPMMs did not provoke any inflammatory response and were excreted safely [84].

Meidahl et al. [85] investigated the nasal delivery of a herpes simplex virus (HSV)-based vector expressing human proenkephalin (SHPE) as a potential treatment for TBI-related pain, using a mild TBI animal model. Two days after injury, rats treated with SHPE exhibited reduced facial allodynia, which was statistically significant from days 4 to 7, with ongoing relief thereafter. A strong presence of human proenkephalin was observed in the trigeminal ganglia of SHPE-treated rats, but not in SHZ.1 control rats [85].

Hypoxia-induced neuronal cell death is strongly associated with the activation of microglial cells [86]. The intranasal delivery of microglial receptor Mac-1 siRNA, combined with invivofectamine reagent, reduces neurodegeneration in the prefrontal cortex and hippocampus, and alleviates working memory deficits in mice with hypoxic brain injury [87]. The miRNA-124 is often used to evaluate neuroprotection and functional recovery after cerebral stroke due to its role in neuronal differentiation, maturation, and survival [88,89]. This miRNA was administered intranasally in rat models of middle cerebral artery occlusion (t-MCAO) with ischemic brain injury. The results showed that nasal administration of miRNA-124 had significant effects on ischemic brain injury, and RVG29-PEG-PLGA/miRNA-124 could be a promising method for treating neurodegenerative diseases [90].

Given its potential, exosome-based nasal administration has been explored as an alternative to cell transplantation in treating spinal cord injury (SCI). Guo et al. [91] studied intranasal administrations of exosomes derived from mesenchymal stem cells (MSC-Exo) loaded with phosphatase and tensin homolog (PTEN) siRNA (ExoPTEN) in rats with complete SCI [91]. PTEN is present in neurons and regenerating axons, where it plays a crucial role in corticospinal neuron regeneration by reducing cytoplasmic mammalian target of rapamycin (mTOR) activity [92]. The results indicated that intranasal ExoPTEN decreases PTEN expression, which encourages axonal regeneration and the development of new blood vessels. Furthermore, this treatment partly enhances both structural and electrophysiological functions and, most importantly, results in significant functional recovery in rats with complete SCI injuries [91].

### 5.2. Neurodegenerative Disorders

Alzheimer’s disease (AD) is a progressive neurodegenerative disorder characterized by amyloid-β deposition and cognitive decline [92,93,94,95]. Kim et al. [93] demonstrated that nasal immunization of a young AD mouse model with an adenovirus vector encoding 11 repeats of Aβ1-6 fused to the receptor-binding domain (Ia) of *Pseudomonas* exotoxin A (AdPEDI-(Aβ1-6)11) triggered the production of anti-amyloid antibodies, decreased Aβ accumulation, enhanced learning and memory, and elevated IL-10 expression [93].

Evidence indicates that hypoxia heightens oxidative stress damage and plays a role in the initiation and development of AD [94,95]. Intranasal delivery of a lentiviral vector encoding human nuclear factor erythroid 2-related factor 2 (NRF2) improved the weak antioxidant response caused by hypoxia, reduced Aβ deposition, and enhanced spatial memory function [96]. MiRNA-based therapies hold significant promise for neurodegenerative diseases, with miR-132 showing potential in alleviating symptoms of AD [66,97,98]. Recently, Su et al. [66] created WGA-modified PEG-PLA nanoparticles (WGA-NPs) loaded with miR-132 for nasal administration in mouse and rat models of AD with ischemic brain injury. Their assessment, which measured infarct size, neuronal death, and neuroinflammation, indicated that nasal delivery of miR-132 could offer strong neuroprotection in AD and cerebral ischemia [66].

Parkinson’s disease (PD) is a progressive neurodegenerative disorder caused by the death of dopaminergic neurons in the substantia nigra and abnormal accumulation of α-synuclein in the form of Lewy bodies and Lewy neurites [99,100]. Alarcón-Arís et al. [101] showed that siRNA and antisense oligonucleotide (ASO) molecules conjugated with indatraline (triple monoamine blocker) reduce α-synuclein expression specifically in monoamine neurons, with a high translational value in the treatment of PD. Additionally, this potential treatment improves dopamine and serotonin neurotransmission deficits in PD by enhancing monoamine release and/or reducing their uptake [101]. In another study, intranasal administration of a non-viral vector for human GDNF induced transgene expression in the brain and protected dopamine neurons in a model of PD [102]. GDNF seems to be a potent neurotrophic factor for neurons, promoting their survival and proliferation [103,104].

In Huntington’s disease (HD), a neurodegenerative disorder inherited in an autosomal dominant pattern caused by a mutation in the huntingtin (HTT) gene, the intranasal delivery of chitosan-based nanoparticles containing anti-HTT siRNA decreased HTT mRNA levels by more than 50% in YAC128 transgenic mice [54,105]. Furthermore, Fatani et al. [106] demonstrated that plasmid DNA delivered through 6-O-glycolchitosan nanoparticles induced dose-dependent gene expression mainly in the cerebral cortex and striatum, with minimal systemic exposure [106].

### 5.3. Brain Tumors

In the case of glioblastoma (GBM), nose-to-brain therapies utilizing non-coding RNAs (ncRNA) that modulate tumor gene expression show significant promise for improving treatment outcomes. Researchers developed a grapefruit-derived nanovector hybrid with polyethylenimine (pGNV) for efficient intranasal delivery of miRNA to the brain. Zhuang et al. [107] administered miR-17 intranasally, delivered via folic acid-polyglycine-arginine-vascular (folic acid-pGNV), to tumor-bearing mice. This approach resulted in swift transport of miR-17 to the brain, where it was selectively absorbed by GL-26 tumor cells, leading to a slowdown in tumor growth [107].

Sukumar et al. [108] examined the combined therapeutic effect of delivering two miRNAs to treat GMB (antimiR-21 and miR-100) using theranostic polyfunctional gold-iron oxide nanoparticles (polyGIONs) on their surface. A significant increase in the survival of mice co-treated with T7-polyGIONs loaded with miR-100-antimiR-21 and chemotherapy was observed compared to the untreated control group, mice receiving chemotherapy, or mice treated with polyGIONs-miRNAs alone [108]. Using cationic nanoemulsions as an intranasal CD73-siRNA delivery system, Azambuja et al. [46] showed reducing tumor growth by 60% in glioma-bearing Wistar rats, besides a reduction of 95% in adenosine production in liquor and tumor CD73 expression [47]. Hu et al. [109] developed a lipoplex designed to safely, efficiently, and specifically deliver c-Myc-targeting siRNA (si-c-Myc) to glioma cells through intranasal administration. The lipoplex released siRNA into the cytoplasm within four hours, leading to a significant reduction in c-Myc mRNA and protein levels in the glioma cells [109]. Mice with tumors treated with intranasal HA/DP7-C loaded with VEGF-specific and PLK1-specific siRNA showed reduced tumor growth, extended survival, and smaller tumor sizes [63].

### 5.4. Neuropsychiatric Disorders

In major depressive disorder (MDD), a severe, chronic, and life-threatening disease [110,111], intranasal administration of BDNF fused with cell-penetrating peptides packaged in adenovirus-associated virus (BDNF-HA2TAT/AAV) produced antidepressant effects in the forced swimming test of chronic mild stress (CMS) mice by increasing hippocampal BDNF levels [112]. Subsequently, the BDNF-HA2TAT/AAV construct was employed to deliver BDNF and investigate its antidepressant effects on post-stroke depression (PSD) rats using behavioral and histological methods to uncover the underlying mechanisms [113]. Nasal delivery of BDNF-HA2TAT/AAV selectively increased BDNF mRNA and protein levels in the prefrontal cortex, which may explain its antidepressant effects in the PSD model. Recently, Zhang et al. [114] demonstrated that delivering BDNF-HA2TAT/AAV in rats subjected to single prolonged stress (SPS) elicited anxiety- and depression-like behaviors, observed in the open-field test, elevated plus maze, and forced swim test. These findings also revealed elevated plasma levels of corticosterone, BDNF, and TrkB (tropomyosin receptor kinase B) in the hippocampus [114].

### 5.5. Congenital Metabolic Disorders

Gene therapy delivered through the nose may also help treat congenital CNS disorders. For instance, mucopolysaccharidosis type I (MPS-I), an autosomal recessive lysosomal storage disease caused by a deficiency of α-L-iduronidase (IDUA) leads to the accumulation of glycosaminoglycans (GAGs) and results in various complications in the CNS [115]. In a study by Belur et al. [116], the IDUA gene was delivered intranasally to IDUA-deficient mice using an adeno-associated virus serotype 9 (AAV9) vector. Five months post-treatment, the mice showed restored wild-type levels of IDUA throughout their brains and a notable decrease in GAG accumulation, probably due to enzyme diffusion from the olfactory bulb and nasal epithelium into deeper brain regions. Additionally, neurocognitive assessments indicated that the treated mice performed on par with wild-type controls, demonstrating effective reversal of neurological impairments [116].

Schuh et al. [44] and Vera et al. [45] also investigated nasal gene therapy strategies targeting MPS I. Schuh et al. used a cationic nanoemulsion-based non-viral vector for intranasal *IDUA* gene delivery, which led to a modest increase in IDUA enzymatic activity in multiple organs (lung, kidney, spleen, heart, and brain) and a reduction in GAG levels in serum, urine, tissues, and the brain cortex [44]. Vera et al. employed a non-viral liposomal CRISPR/Cas9 system, nasally delivered, targeting the *IDUA* gene in MPS I mice. Their results showed effective gene editing in both brain and visceral tissues, leading to a significant reduction in GAG accumulation and improvements in behavioral performance, indicating partial correction of the neurological phenotype [45].

## 6. Clinical Perspectives

In vivo studies have shown that nucleic acids delivered through the nose can effectively reach the brain, providing a promising non-invasive treatment option for CNS diseases [42,117]. However, despite promising preclinical results and advancements, the field remains in its early stages; none have achieved clinical application, with many challenges still to overcome. Current studies mainly focus on demonstrating the transport of biomolecules to the brain; however, a limited understanding exists of the mechanisms behind nose-to-brain administration, including the specific transport pathways and absorption processes, as well as the lack of standardized methods for assessing pharmacokinetics in humans. Additionally, factors such as scalability, formulation stability, anatomical differences between humans and animal models, and the development of appropriate administration devices must be considered. This gap hampers the rational design of more effective administration strategies. Translating these findings into clinical practice also involves optimizing administration efficiency, long-term safety, dosing protocols, and immune response management. Furthermore, comprehensive data on biodistribution, off-target effects, and the functional integration of administered genes are essential for progress. To address these challenges, collaboration among industry, academia, and regulatory agencies is crucial [118].

## 7. Conclusions

Nose-to-brain drug delivery is a promising strategy to overcome the BBB, allowing non-invasive and direct access to specific neural pathways in the brain. This approach enhances drug availability in the CNS while decreasing systemic exposure, thus enhancing the therapeutic outcomes. The variety of delivery systems, ranging from viral vectors (e.g., AAV, HSV) to non-viral nanostructures such as lipid nanoparticles, polymeric nanoparticles, liposomes, and nanoemulsions, along with advanced gene therapies, offers versatile tools tailored for CNS disorders. Additionally, innovative techniques such as nanotechnology-based mucoadhesive strategies, modified peptides, and depot implants have shown potential to enhance nasal residence time, targeting efficiency, and therapeutic outcomes. Overall, these preclinical findings demonstrate that the intranasal delivery of nucleic acid-based therapies enables widespread and sustained transgene expression across various brain regions, effectively bypassing the BBB in a minimally invasive manner. This approach demonstrates potential for a wide range of CNS conditions, including neuropsychiatric disorders, brain injury, tumors such as glioblastoma, neurodegenerative diseases (e.g., AD, PD, HD), and congenital metabolic disorders (e.g., MPS I). However, despite encouraging preclinical results, the field remains in early development stages; none of these approaches have yet reached clinical use, with many challenges still to be addressed. From a future perspective, continued research focused on innovative delivery strategies, mechanistic understanding, and clinical trials to validate safety and effectiveness will be essential to advance nose-to-brain gene therapy toward clinical use for CNS disorders.

## Figures and Tables

**Table 1:** Summary of key findings from the literature on nanostructures and biomaterials used for nose-to-brain nucleic acids delivery.

Nucleic Acid	Gene	Delivery Strategy	Key Findings	**Ref.**
DNA plasmid	IDUA (alpha-L-iduronidase)	Nanoemulsions (DOPE, DOTAP, MCT, and DSPE-PEG)	The complexes enhanced enzyme activity in vitro and in vivo in a mouse model of MPS I.	[44,45]
	Non-specific pDNA	Cationic nanoparticles (CTAB and Brij 78)	Serum antigen-specific IgG levels increased by 18 to 28 times compared to naked pDNA when pDNA nanoparticles were administered to Balb/C mice.	[48]
mRNA	GFP-mRNA	Liposomes (DPPC, DOTAP, and cholesterol)	In vivo biodistribution revealed 15% higher GFP-mRNA expression in the brain compared to the naked mRNA group.	[47]
miRNA	miR-132	mPEG-PLA nanoparticles	The distribution of WGA-NPs-miR132 in the APP/PS1 mouse model and MCAO rats was notably improved in the brain, demonstrating therapeutic effects, reduced nasal ciliary clearance, and better targeting to neurons.	[66]
ODN	PTO	Microparticles coated with PCP-Cys	Microparticles reduced clearance from the nasal cavity, increased contact time with the nasal mucosa, and offered high stability, thereby improving ASO permeation and controlling release.	[51,52]
siRNA	CD73	Nanoemulsions (DOTAP, MCT, and lecithin E-80)	CD73 was silenced in vitro and in vivo, resulting in a 60% decrease in tumor size.	[46]
	Beclin1	Cationic PEI-siBeclin1 nanoplexes	siRNA was detected in the glial cells of the prefrontal cortex and the cytoplasm of neurons at 4 and 24 h post-administration, with a notable 65% reduction in protein levels and no signs of toxicity.	[49]
	32-P labeled siRNA	Thermosensitive mucoadhesive gels containing siRNA dendriplexes	Decreased degradation by RNases and enhanced endocytic uptake.	[59]
	HTT	Nanoparticles with chitosan	Four nanocarrier formulations were found to reduce HTT mRNA expression by at least 50%.	[54]
	VEGF or PLK1	Multifunctional core–shell nanomicelles coated with hyaluronic acid (HA) and encapsulating the cell-penetrating peptide DP7-C	Successfully delivering the formulation to GL261 tumor-bearing mice resulted in tumor growth inhibition, longer survival times, and smaller tumor volumes.	[63]
	Raf-1	Nanomicelles with PEG-PCL conjugated with the cell-penetrating peptide Tat	Nasal administration of these nanomicelles to rats resulted in a ten-fold increase in siRNA levels in the brain.	[64]
	BACE1	Solid lipid nanoparticles with chitosan and RVG-9R, a cell-penetrating peptide	In vitro tests using the Caco-2 cell line demonstrated that formulations exhibited increased permeability, while those coated with chitosan showed decreased passage through the cell layer.	[69]
	BACE1 and Caspase-3	Exosomes combined with BAP	The exosomes significantly increased brain accumulation of Cy5-labeled siRNA, and treated mice outperformed saline controls in spatial learning and memory tests.	[72]

## Data Availability

Not applicable.
